# High mean arterial pressure target to improve sepsis-associated acute kidney injury in patients with prior hypertension: a feasibility study

**DOI:** 10.1186/s13613-021-00925-2

**Published:** 2021-09-22

**Authors:** Antoine Dewitte, Aurore Labat, Pierre-Antoine Duvignaud, Gauthier Bouche, Olivier Joannes-Boyau, Jean Ripoche, Gilles Hilbert, Didier Gruson, Sébastien Rubin, Alexandre Ouattara, Alexandre Boyer, Christian Combe

**Affiliations:** 1 CHU Bordeaux, Department of Anaesthesia and Critical Care, Magellan Medico-Surgical Centre, F-33000 Bordeaux, France; 2 Univ. Bordeaux, CNRS, UMR 5164, ImmunoConcEpT, F-33000 Bordeaux, France; 3 CHU Bordeaux, Department of Nephrology-Transplantation-Dialysis-Apheresis, Hôpital Pellegrin, F-33000 Bordeaux, France; 4 The Anticancer Fund, 1853 Strombeek-Bever, Belgium; 5 Univ. Bordeaux, INSERM, UMR 1026, F-33000 Bordeaux, France; 6 CHU Bordeaux, Medical Intensive Care Unit, Hôpital Pellegrin, F-33000 Bordeaux, France; 7 Univ. Bordeaux, INSERM, UMR 1034, Biology of Cardiovascular Diseases, F-33600 Pessac, France

**Keywords:** Acute kidney injury, Septic shock, Intensive care, Blood pressure, Norepinephrine, Kidney concentrating ability

## Abstract

**Background:**

The optimal mean arterial pressure (MAP) in cases of septic shock is still a matter of debate in patients with prior hypertension. An MAP between 75 and 85 mmHg can improve glomerular filtration rate (GFR) but its effect on tubular function is unknown. We assessed the effects of high MAP level on glomerular and tubular renal function in two intensive care units of a teaching hospital. Inclusion criteria were patients with a history of chronic hypertension and developing AKI in the first 24 h of septic shock. Data were collected during two 6 h periods of MAP regimen administered consecutively after haemodynamic stabilisation in an order depending on the patient's admission unit: a high-target period (80–85 mmHg) and a low-target period (65–70 mmHg). The primary endpoint was the creatinine clearance (CrCl) calculated from urine and serum samples at the end of each MAP period by the UV/P formula.

**Results:**

26 patients were included. Higher urine output (+0.2 (95%:0, 0.4) mL/kg/h; *P* = 0.04), urine sodium (+6 (95% CI 0.2, 13) mmol/L; *P* = 0.04) and lower serum creatinine (− 10 (95% CI − 17, − 3) µmol/L; *P* = 0.03) were observed during the high-MAP period as compared to the low-MAP period, resulting in a higher CrCl (+25 (95% CI 11, 39) mL/mn; *P* = 0.002). The urine creatinine, urine–plasma creatinine ratio, urine osmolality, fractional excretion of sodium and urea showed no significant variation. The KDIGO stage at inclusion only interacted with serum creatinine variation and low level of sodium excretion at inclusion did not interact with these results.

**Conclusions:**

In the early stage of sepsis-associated AKI, a high-MAP target in patients with a history of hypertension was associated with a higher CrCl, but did not affect the kidneys' ability to concentrate urine, which may reflect no effect on tubular function.

**Supplementary Information:**

The online version contains supplementary material available at 10.1186/s13613-021-00925-2.

## Background

Acute kidney injury (AKI) is a common clinical problem affecting approximately 50% of intensive care patients [1]. Sepsis is its main cause in this setting [2]. Traditional hemodynamic management of sepsis-associated AKI focuses on the prevention of hypoperfusion by optimizing blood pressure to maintain renal perfusion pressure and thus glomerular filtration rate (GFR), primarily through fluid resuscitation and administration of vasopressor drugs. However, optimizing blood pressure to limit kidney damage is a daily challenge for intensivists, especially since the optimal mean arterial pressure (MAP) target remains a subject of debate [3–7]. In patients with septic shock, the Surviving Sepsis Campaign guidelines recommend an initial target MAP of 65 mmHg. It is also highlighted that when a better understanding of any patient’s condition is obtained, the MAP target should be individualized to the pertaining circumstances as it may be too low for certain patients [3]. In particular, the threshold for renal autoregulation may be higher in patients with atherosclerosis and/or previous hypertension than in young patients without cardiovascular comorbidity. European expert recommendations suggest higher MAP target in septic shock patients with history of hypertension and in patients that show clinical improvement with higher blood pressure [4].

On the other hand, there may be a risk of excessive vasoconstriction at higher MAP target requiring higher norepinephrine infusion rates, particularly in cases of sepsis-associated AKI where the pathophysiological mechanisms are complex [5]. Sepsis-associated AKI is characterized at an early stage by increased renal blood flow and decreased renal vascular conductance, resulting in redistribution of intrarenal blood flow and reduced medullar perfusion and oxygenation [1, 6]. Restoration of blood pressure by norepinephrine infusion improves renal perfusion pressure but may further reduce renal medullary perfusion at high concentrations [6]. A higher MAP target could then improve blood pressure on the glomeruli, located in the renal cortex, without benefiting renal tubular function dependent on medullary perfusion.

The objective of this study was to analyse the effects of a high-MAP target on renal glomerular and tubular function in critically ill septic patients with a history of chronic hypertension.

## Methods

### Patients and setting

During a 12-month study period (August 2016–July 2017), we included patients with a history of chronic hypertension and developing AKI at any KDIGO stage in the first 24 h of septic shock in two intensive care units (ICU) of Bordeaux University Hospital (one medico-surgical ICU including pulmonary or abdominal surgery and one medical ICU). Exclusion criteria were pregnancy, age ≤ 18 years, obstructive renal disease, AKI from an obstructive or suspected cause other than sepsis (e.g., toxic), severe chronic kidney disease defined based on a known eGFR < 30 mL/min/1.73 m^2^, renal replacement therapy (RRT) or anuria at the time of inclusion, and a presumed life expectancy < 24 h. Approval for this study was obtained from our institutional review board (*Trial registration: DC 2016/81, Comité de Protection des Personnes Sud-Ouest et Outre Mer III, France; *http://www.cpp-soom3.u-bordeaux2.fr/*; Registered June 2016*). All patients or their family agreed to participate in the study.

### Definitions

Patients were defined as having chronic hypertension when they were known to be hypertensive in their past medical history with at least one antihypertensive treatment in their usual medication regimen. Septic shock was defined according to the sepsis-3 definition [7]. AKI was defined according to the Kidney Disease Improving Global Outcomes (KDIGO) classification on the criteria of urine output (UO) and serum creatinine (sCr) [8]. The baseline sCr was determined by calling the referring doctor or by analysing the patient’s medical records within the prior 3 months.

### Procedures

The attending physician treated patients in accordance with the recommendations of the Surviving Sepsis Campaign after admission to ICU [3]. Initial management included fluid challenges to achieve a minimum of 30 mL/kg crystalloids and avoid excessive vasoconstriction in hypovolemic patients. Fluid administration was continued if there was haemodynamic improvement based on dynamic criteria (e.g., change in stroke volume). Haemodynamic was continuously monitored for all patients by an arterial line and repeated echocardiography. The administration of norepinephrine was performed to reach an initial MAP target of 65 mmHg.

**Fig. 1:**
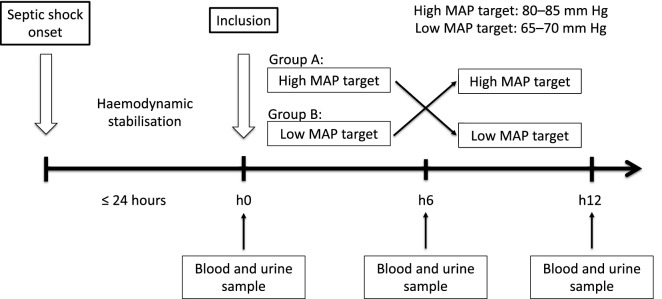
Study design

After haemodynamic stabilisation defined as a 3 h stable or decreased dose of norepinephrine without a need for fluid loading, the MAP target was challenged in accordance with the recommendations [4, 9]. Patients were studied during two consecutive 6-h periods of MAP regimen administered consecutively in an order that depended on the ICU to which the patient was admitted: a group A with a high target period with MAP of 80–85 mmHg followed by a low-target period with MAP of 65–70 mmHg and a group B with a low MAP target period followed by a high MAP target period (Fig. 1). The order of assignment of the MAP regimens depended on the two participating ICU and the treatment was not blinded to the investigators or participants. Norepinephrine was titrated by the attending physician and the ICU nurses to achieve the MAP target according to unit protocols.

During the high-MAP target period, a reduction in vasopressor doses to maintain an MAP of 65 to 70 mmHg was recommended if any of the prespecified serious adverse events that were potentially related to an increased rate of vasopressor infusion occurred. These events clinically relevant were bleeding, rhythm disorders, suspicion of myocardial infarction, mesenteric ischemia or distal-limb ischemia.

### Data collection

All data, including hourly UO, were collected over a period of 6 h for each MAP target. Patient monitoring software (Metavision; iMDSoft, Wakefield, MA, USA) was used to continuously record all variables with a time interval of 1 min. The data were automatically averaged for each point analyzed.

### Endpoints

The primary endpoint of this study was the GFR estimated by calculating creatinine clearance (CrCl) with the UV/P formula [9]. CrCl was determined from a urine sample obtained from the collection of the last hour of each MAP period. Urine and blood samples were collected simultaneously at inclusion and at each change in MAP target (Fig. 1). The other secondary endpoints were the sCr, urine creatinine (UCr), UO, urine sodium (UNa), serum osmolality, urine osmolality, proteinuria, urine-to-plasma creatinine ratio, fractional excretion of sodium (FeNa) and fractional excretion of urea (FeU) variations from low to high-MAP period. The occurrence of adverse events was also analysed.

### Statistical analyses

We estimated that at least 20 patients with AKI would be needed in this study to have 80% power to detect a 20% difference in CrCl between the two periods at a two-sided alpha level of 0.05. This calculation was based on the assumption that the standard deviation of the difference between the two CrCl values for the same patient would be 30%. Quantitative parameters are reported as their mean (standard deviation) or median [interquartile range] as appropriate and qualitative parameters are expressed as numbers (percentages). Baseline characteristics were compared using the χ^2^ test or Fisher’s exact test as appropriate. Continuous variables were compared using the Mann–Whitney *U* test. We performed a multivariate repeated measures analysis of variance (MANOVA) to compare the primary and secondary endpoints between the inclusion and the low MAP regimen and between the low and the high MAP regimen, including the order of assignment of MAP regimes and the KDIGO stage at inclusion as factors. Analysis of the interaction of pre-inclusion ACE inhibitor treatment, time from initiation of norepinephrine to inclusion, level of sodium excretion at inclusion and norepinephrine dose to achieve a high-MAP target on the variables studied was also assessed using multivariate repeated measures analysis of variance (MANOVA). All tests were two-sided with an alpha level of 0.05. Statistical analyses were performed using SAS version 9.3 (SAS Institute, Cary, NC, USA) and GraphPad Prism version 6.00 (GraphPad Software, La Jolla, CA, USA).

## Results

### Study population

**Fig. 2:**
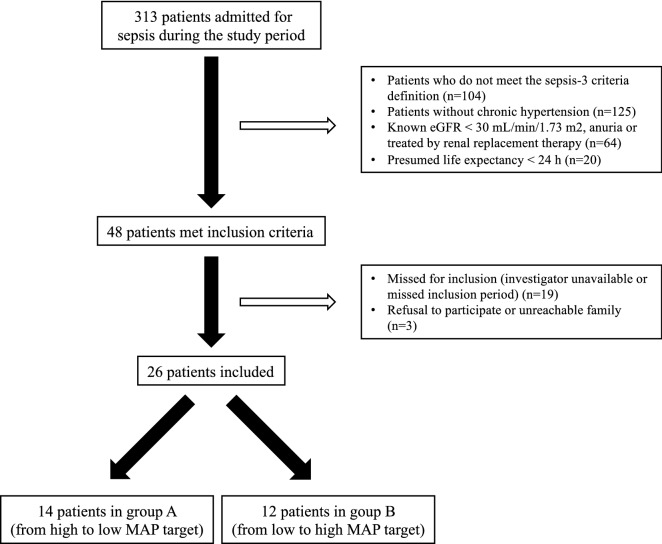
Flow chart of the study

Twenty-six patients were included in the study. The flow chart and the characteristics of the patients are shown in Fig. 2 and Table 1. All antihypertensive treatments were stopped at the onset of septic shock. No patients received diuretic therapy after admission to the ICU. All patients were mechanically ventilated. The median time from initiation of norepinephrine to inclusion was 18 [12–24] h. The fluid balance was positive before inclusion with a median of 54 [38–93] mL/kg since ICU admission. No patients died during the study (Table 2). Six patients were treated with RRT after the study period with a median time from admission to initiation of RRT of 93 [16–144] h.

**Table 1:** Baseline characteristics of patients (*n* = 26)

Characteristics	All patients (*n* = 26)	Group A: from high to low MAP target (*n* = 14)	Group B: from low to high MAP target (*n* = 12)	*P* value
Demographic characteristics				
Age (year)	69 [61–75]	68 [61–75]	69 [60–75]	0.7
Male	21 (80)	11 (79)	10 (83)	0.8
Weight (kg)	80 [65–94]	80 [65–95]	82 [59–100]	0.8
Body Mass Index	27 [24–32]	28 [24–31]	26 [22–32]	0.8
Reason for admission				0.05
Medical	10 (38)	3 (21)	7 (58)	
Surgical	16 (62)	11 (79)	5 (42)	
Antihypertensive treatment				0.4
Calcic inhibitor	13 (50)	8 (57)	5 (42)	
Angiotensin converting enzyme inhibitor	10 (39)	5 (36)	9 (75)	
Beta-blockers	11 (42)	5 (36)	6 (50)	
Diuretic	2 (8)	1 (7)	1 (8)	
Comorbidities				0.8
Coronaropathy	7 (27)	4 (29)	3 (25)	
Valvular disease	3 (12)	1 (7)	2 (17)	
Rhythmic disease	8 (31)	5 (36)	3 (25)	
Heart failure	4 (15)	3 (21)	1 (8)	
Peripheral arterial disease	1 (4)	1 (7)	0 (0)	
Ischemic stroke	1 (4)	1 (7)	0 (0)	
Chronic kidney failure	4 (15)	3 (21)	1 (8)	
Respiratory disease	8 (31)	4 (29)	4 (33)	
Diabetes mellitus	10 (39)	6 (43)	4 (33)	
Neoplastic disease	10 (39)	7 (50)	3 (25)	
Smoking	7 (27)	4 (29)	3 (25)	
Prior kidney function				
Baseline sCr (µmol/L)	71 [53–90]	68 [41–90]	73 [60–90]	0.6
Baseline eGFR^a^ (mL/min/1.73 m^2^)	92 [75–104]	92 [74–114]	89 [76–97]	0.6
Characteristics at inclusion				
SOFA score	9 [7–13]	9 [6–10]	9 [7–14]	0.5
SAPS II score	52 [36–62]	48 [34–53]	61 [41–73]	0.04
Time from initiation of norepinephrine to inclusion (hours)	18 [12–24]	18 [16–24]	16 [10–23]	0.3
Baseline KDIGO stage				0.6
1	9 (35)	4 (29)	5 (42)	
2	13 (50)	7 (50)	6 (50)	
3	4 (15)	3 (21)	1 (8)	
sCr (µmol/L)	124 [75–192]	143 [116–206]	105 [69–155]	0.1
MAP (mmHg)	71 [65–72]	71 [69–73]	67 [65–73	0.2

Data are expressed as the median [interquartile range] or number (%)

^a^Calculated using the CKD-EPI formula

^b^Calculated using the UV/P formula

ARB: angiotensin II receptor blocker; sCr: serum creatinine; SOFA: Sepsis-related Organ Failure Assessment; SAPS II: simplified acute physiology score II; eGFR: estimated glomerular filtration rate; MAP: mean arterial pressure; ICU: intensive care unit

**Table 2:** Patient outcomes

Outcomes	All patients (*n* = 26)	Group A: from high to low MAP target (*n* = 14)	Group B: from low to high MAP target (*n* = 12)	P value
Renal replacement therapy	6 (24)	4 (31)	2 (17)	0.4
Time from diagnosis of septic shock to initiation of renal replacement therapy (hours)	93 [16–144]	93 [27–136]	81 [18–144]	0.8
ICU, length of stay (days)	15 [13–33]	22 [15–57]	13 [10–25]	0.07
Hospital, length of stay (days)	30 [25–48]	33 [21–72]	29 [23–44]	0.7
ICU Mortality	10 (38)	4 (29)	6 (50)	0.5
Hospital Mortality	11 (42)	5 (36)	6 (50)	0.5

Data are expressed as the median [interquartile range] or number (%)

### Vasopressor use and fluid balance between low- and high-MAP periods

**Fig. 3:**
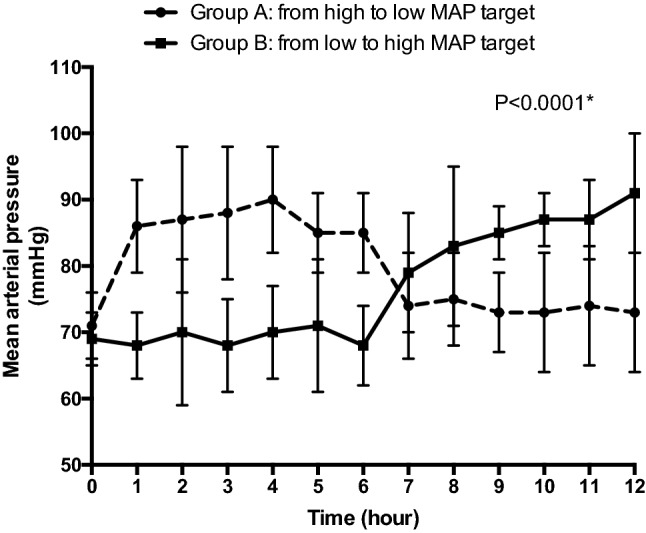
MAP evolution during the high-MAP target period and the low-MAP target period. *P values for the within-subject comparison of MAP using a multivariate repeated-measures analysis of variance (MANOVA)

All patients were receiving norepinephrine during the study period. MAP was significantly different between the low-MAP and high-MAP periods (72 [68–78] vs. 85 [83–89] mmHg in group A and 68 [66–72] vs. 85 [82–89] in group B; *P* < 0.0001) (Fig. 3). To obtain a higher MAP target, patients received higher doses of norepinephrine (0.3 [0.1–0.5] vs. 0.5 [0.3–0.6] µg/kg/min in group A and 0.3 [0.2–0.6] vs. 0.5 [0.4–0.6] µg/kg/min in group B; *P* < 0.0001). No patients received fluid loading during the protocol period. There was no difference in fluid balance during the low- and high-MAP target periods (4 [− 1 to 6] vs. 6 [4–11] mL/kg in group A and 9 [4–11] vs. 8 [4–16] mL/kg in group B; *P* = 0.6). There was no significant interaction of the group or the KDIGO stage at inclusion on these results.

### Endpoints

**Fig. 4:**
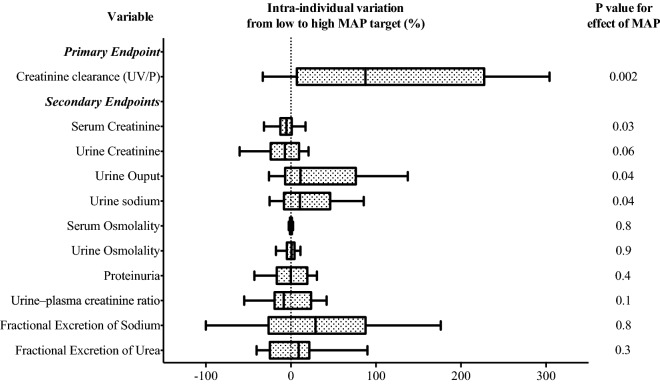
Primary and secondary endpoints. Intra-individual variations in blood and urine parameters from low to high-MAP target period, expressed as a percentage. GFR was estimated by the UV/P (Creatinine Clearance). *P* values for the within-subject comparison of MAP using a multivariate repeated-measures analysis of variance (MANOVA)

The intra-individual variations of the studied parameters from low- to high-MAP target and their values at inclusion are presented in Table 3. No parameters differ significantly between their value at inclusion and the low-MAP target period (Additional file 1: Figure S1). The CrCl was higher during the high-MAP period compared to the low-MAP period with an intra-individual percentage variation of 88 [7–227] % (*P* = 0.002) (Fig. 4). The high-MAP period was associated with a significant intra-individual increase of UO (11 [− 7 to 76] %; *P* = 0.04) and UNa (11 [− 8 to 46] %; *P* = 0.04) and a decrease of sCr (− 5 [− 12 to 1] %; *P* = 0.03) compared to the low-MAP period. There was no significant variation in uCr, urine osmolality, serum osmolality, FeNa and FeU between the low and the high-MAP period.

**Table 3:** Blood and urine parameters according to mean arterial pressure regimen (*n* = 26)

Variables	At inclusion	Low-MAP target period	High-MAP target period	Mean intra-individual difference (95% CI)^a^	*P* values for Group-MAP interaction	P values for effect of MAP
Primary endpoint						
Creatinine clearance (mL/mn)^a^	34 (24)	32 (31)	58 (41)	25 (11, 39)	0.1	0.002
Secondary endpoints						
Serum creatinine (µmol/L)	142 (80)	150 (85)	141 (80)	− 10 (− 17, − 3)	0.5	0.03
Urine creatinine (mmol/L)	7.3 (4.6)	7.7 (6.1)	6.5 (4.1)	− 1.1 (− 2.2, 0.05)	0.2	0.06
Urine output (mL/kg/h)	0.7 (0.5)	0.8 (0.6)	1 (0.7)	0.2 (0, 0.4)	0.3	0.04
Urine sodium (mmol/L)	44 (30)	40 (26)	59 (26)	6 (0.2, 13)	0.8	0.04
Serum osmolality (mOsm/kg)	308 (18)	307 (18)	306 (15)	− 0.1 (− 2.1, 1.9)	0.2	0.8
Urine osmolality (mOsm/kg)	436 (141)	456 (179)	433 (126)	3 (− 18, 25)	0.1	0.9
Proteinuria (g/L)	1.2 (0.8)	1.1 (0.6)	1.2 (0.7)	0.07 (− 0.1, 0.3)	0.9	0.4
Urine–plasma creatinine ratio	75 (72)	78 (86)	70 (71)	− 7 (− 21, 7)	0.8	0.1
Fractional excretion of sodium	0.9 (1.1)	1.1 (1.7)	1.2 (1.4)	0.02 (− 0.2, 0.3)	0.8	0.8
Fractional excretion of urea	31 (17)	32 (13)	36 (17)	2.2 (− 1.7, 6.1)	0.2	0.3

Data are expressed as the mean (SD) and mean individual difference (IC95%) from low- to high-MAP target period

*P* values for the within-subject comparisons between the low and the high-MAP target period a multivariate repeated-measures analysis of variance (MANOVA) including the order of assignment of MAP regimes and the KDIGO stage at inclusion as factors

^a^Calculated using the UV/P formula

MAP: mean arterial pressure; KeGFR: kinetic estimated glomerular filtration rate

**Fig. 5:**
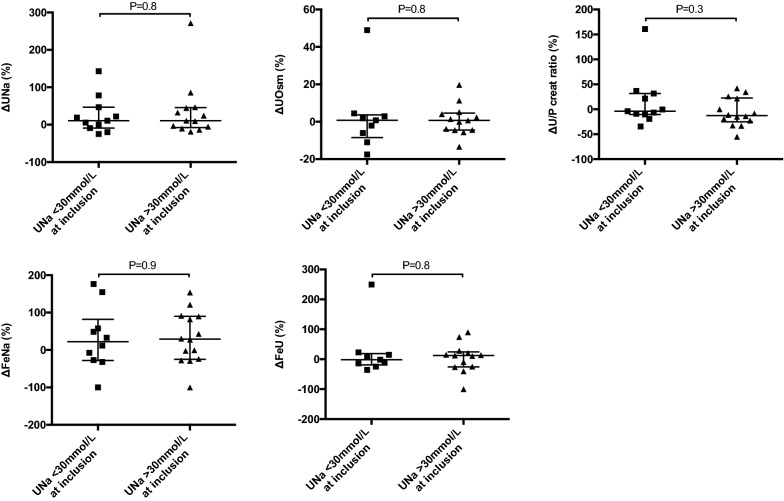
Urine concentration ability according to the natriuresis at inclusion. Intra-individual variations of urine sodium (UNa), urine osmolality (UOsm), urine-to-plasma creatinine ratio (U/P creat ratio), fractional excretion of sodium (FeNA) and fractional excretion of urea (FeU) from low to high-MAP target period according to UNa at inclusion, expressed as a percentage

Urine concentration ability according to the natriuresis at inclusion are shown in Fig. 5. Patients with UNa < 30 mmol/L at inclusion had no significant difference in intra-individual variations of UNa, UOsm, urine-to-plasma creatinine ratio, FeNA and FeU from low to high-MAP target period compared to those with UNa > 30 mmol/L at inclusion.

### Interaction analysis

The order of assignment of MAP regimens did not significantly affect the effects of MAP on the variables analysed (Table 2 and Additional file 1: Table S1). Analysis of the interaction of the KDIGO stage at inclusion only showed a significant interaction on the variation of sCr between the low and high-MAP target period (*P* value for interaction = 0.04) (Additional file 1: Table S2). Treatment with ACE inhibitors prior to inclusion, time from initiation of norepinephrine to inclusion > 18 h, NaU at inclusion < 30 mmol/L and norepinephrine dose required to achieve a high MAP target > 0.5 µg/kg/min also did not interact with the effects of the high vs. low-MAP period on CrCl or any of the secondary endpoints.

### Adverse events

Higher level of MAP target was not associated with more adverse events: seven patients (27%) presented with rhythm disorders (atrial fibrillation) regardless of MAP level. None of the patients suffered from acute coronary events, bleeding, mesenteric ischemia or cutaneous necrosis between the low- and high-MAP target period (troponin level [0.005–0.145] ng/mL vs. 0.03 [0–0.11] ng/mL, *P* = 0.4). Lactate and pH remained similar between the low- and high-MAP target period (1.4 [1.1–1.2] mmol/L vs. 1.4 [1–1.9] mmol/L; *P* = 0.7 and 7.40 [7.32–7.44] vs. 7.39 [7.33–7.44], respectively; *P* = 0.13).

## Discussion

The main finding of this study is that a high-MAP target of 80–85 mmHg compared to MAP of 65–70 mmHg in septic patients with AKI and prior hypertension is associated with increased UO, UNa and decreased sCr, resulting in increased glomerular function as assessed by the UV/P formula. Conversely, a high-MAP regimen does not affect the kidneys' ability to concentrate urine, with no variation in urinary osmolality, urine-to-plasma creatinine ratio and fractional excretion of sodium or urea, which may reflect no effect on tubular function.

The evaluation of renal function is complex in critically ill patients. Serum creatinine is traditionally used, because it is freely filtered into the glomerulus, with a small proportion being secreted along the tubule. The recommended formula for estimating GFR is UV/P, because it has the potential advantage that it can be used in the absence of a stable state [10]. However, its main limitations are that many factors influence sCr, in particular the patient's volume of distribution [11] and the proportion of tubular creatinine secretion, which remains unpredictable and depends on the relative increase in sCr to the patient's baseline creatinine [10]. However, the kidney has many other functions that are difficult to assess in AKI, including tubular transport. The ability of loop diuretics that are active on the renal tubule to induce natriuresis has, for example, been used to predict the development and severity of AKI and its prognosis [12]. To our knowledge, no studies have estimated the impact of higher pressure regimen on tubular function in sepsis-associated AKI.

The effects of MAP level on AKI have been investigated in numerous studies, showing an improvement in UO with a MAP target between 65 and 75 mmHg [13]. In the SEPSISPAM trial, 778 patients with septic shock were randomly treated with a low (65–70 mmHg) vs. high (80–85 mmHg) MAP target [14]. The authors demonstrated less renal failure, as defined by the doubling of plasma creatinine (38.8% vs. 52.0%, respectively, *P* = 0.02) in patients with previous hypertension treated with a higher MAP target and a decrease number of patients requiring RRT. Conversely, the 65-trial comparing permissive hypotension to usual care in patients 65 years of age or older receiving vasopressors for vasodilatory hypotension did not demonstrate an increase in the use of RRT in patients with chronic hypertension randomized to a lower MAP target group [15]. Legrand et al. also did not observe an association between most systemic hemodynamic parameters, including MAP and cardiac output, and sepsis-associated AKI [16]. Our results confirm that in patients with sepsis-associated AKI and chronic hypertension, a higher MAP target is associated with a significant increase in UO and a decrease in sCr, resulting in better CrCl. The observed increase in CrCl may not be considered as the only result of the increase in UO (e.g., single doses of diuretics have no significant effect on CrCl [17]), but could rather be the consequence of a residual level of glomerular filtration function when capillary pressure increases during a high-MAP regimen [18, 19]. The stage of AKI interacted with variations in sCr in our study, possibly due to higher sCr values in patients with the most severe renal injury. More originally, we showed no variation in the ability to concentrate urine at a high-MAP regimen. Increased renal perfusion could, for instance, have curbed sodium reabsorption, but patients with low sodium excretion at inclusion showed no significant change in their ability to concentrate urine at high-MAP regimen compared to those with higher sodium excretion at inclusion. Furthermore, proximal tubular creatinine secretion accounts normally for 10–20% of the total creatinine clearance but increases to 50% in chronic kidney disease (CKD) when GFR falls [20]. The high-MAP regimen in our study did not result in a significant increase in uCr, which may also reflect no effect on tubular secretion of creatinine.

This study has several limitations. First, this study should be considered exploratory and observational as the crossover was only performed at the individual level and not at the level of the grouping unit without randomisation. A carry-over effect remains, therefore, possible in this rapidly evolving disease without being able to assume that the patients had returned to their initial state before the application of the next MAP regimen. However, changes in creatinine clearance are described as rapid in AKI, with a period of approximately 7 h to reach a 100% increase when the baseline SCr is 88 µmol/L at a constant rate of creatinine production of 60 mg/h and a complete cessation of CrCl [21]. A washout period was also possible on the urine sample, since it was collected on the last hour collection. The time to assess a change in renal tubular function is not known for this clinical setting either, but response to renal tubular function tests is often observed within hours in nephrology studies [22]. Second, patients were included after the initial resuscitation of septic shock, i.e., after the crucial period for the onset and severity of AKI. The inclusions may then have been too delayed for the MAP-targeted norepinephrine regimen to have a significant impact on tubular function. Patient evolution prior to admission to the ICU may also have influenced our results and the time period for a potential impact of higher MAP regimen on renal function and its lasting effect is unknown. The haemodynamic stabilisation period defined by a stable or decreased dose of norepinephrine for 3-h fluid loading could also be debated. This delay was chosen to allow sufficient time to reach an optimised volume status before changing doses of norepinephrine, but without excessively delaying the possible effect of the change in pressure regimen on renal function. Estimation of GFR by calculation of a CrCl from a urine sample may be another limiting factor as it may not be representative of urine production over a longer period of time [9]. However, our analysis at an intra-individual level in combination with an MAP regimen administered consecutively in two different orders may avoid other biases related to the normal evolution of sepsis-associated AKI, given the great diversity of septic patients, including the patient's creatinine generation rate, the volume of distribution of creatinine, and dynamic changes over time as well as “renal reserve” of patients.

The pathophysiological mechanisms underlying sepsis-associated AKI are still a matter of debate, but it has been demonstrated that AKI occurs during hyperdynamic sepsis with increased total renal blood flow [5, 23]. Several mechanisms have been proposed to play a role, including tissue hypoxia, changes in microcirculation, venous congestion and mechanisms independent of haemodynamic impairment, such as inflammation and oxidative stress. Beyond the filtration function of the kidney, tubular transport is a determining factor in its oxygen consumption and evidence is now accumulating that places the tubular system at the center of AKI pathophysiology and recovery in established sepsis [24]. In addition, recent findings suggest that treatment with norepinephrine decreased medullary tissue oxygen tension by half and decreased medullary perfusion, region in which the renal tubules are inserted [6]. The increase in GFR during norepinephrine infusion may also increase sodium delivery to tubular elements within the medulla, and thus utilization of oxygen for sodium reabsorption, which could contribute to the observed medullary hypoxia [25]. Whether the risk of medullary hypoxia associated with high doses of norepinephrine combined to pressure induced glomerular injuries, which is the predominant pathway for nephron loss in CKD [26], have an impact on renal injury and its long-term prognosis is unknown. However, it is now well established that AKI survivors are at high risk of developing CKD, even if their AKI was not severe and their kidney function has recovered on discharge from the ICU [27]. The impact of a high-MAP target on renal tubular function and on the longer term prognosis of sepsis-associated AKI in patients with prior hypertension should be further investigated in larger randomised trials.

## Conclusion

In the early stage of sepsis-associated AKI, a higher MAP target of 80–85 mmHg as compared to a standard MAP target of 65–70 mmHg in patients with prior hypertension was associated with a significant greater glomerular function evaluated by the UV/P formula, but did not affect the kidneys' ability to concentrate urine, which may indicate no effect on tubular function.

## Supplementary Information

**Additional file 1. Table S1.** Blood and urine parameters according to the order of administration of the pressure regimen (*n* = 26). **Table S2.** Mean individual difference in blood and urine parameters according to KDIGO stage (KDIGO 1 patients (*n* = 9) and KDIGO 2-3 patients (*n* = 17)). **Figure S1.** Individual profile of primary and secondary endpoints. Box plot (median values, inter-quartile range) and individual variations of primary and secondary endpoints from inclusion to low MAP target period and from low to high MAP target period (*n* = 26). *P*-values for the within-subject comparison of MAP using a multivariate repeated-measures analysis of variance (MANOVA).

## Data Availability

The data set used during the current study are available from the corresponding author on reasonable request.

## Abbreviations

AKI: Acute kidney injury
CrCl: Creatinine clearance
CKD: Chronic kidney disease
eGFR: Estimated glomerular filtration rate
GFR: Glomerular filtration rate
ICU: Intensive care unit
KDIGO: Kidney Disease Improving Global Outcomes
KeGFR: Kinetic estimated GFR
MAP: Mean arterial pressure
RRT: Renal replacement therapy
sCR: Serum creatinine
uCr: Urine creatinine
UO: Urine output
UNa: Urine sodium
