# Supplementary material for: High mean arterial pressure target to improve sepsis-associated acute kidney injury in patients with prior hypertension: a feasibility study
Source: Ann Intensive Care. 2021 Sep 22;11:139. doi: 10.1186/s13613-021-00925-2 (PMC8458519; doi:10.1186/s13613-021-00925-2)
Supplement: Supplementary file 1 — Additional file 1. Table S1. Blood and urine parameters according to the order of administration of the pressure regimen (n = 26). Table S2. Mean individual difference in blood and urine parameters according to KDIGO stage (KDIGO 1 patients (n = 9) and KDIGO 2-3 patients (n = 17)). Figure S1. Individual profile of primary and secondary endpoints. Box plot (median values, inter-quartile range) and individual variations of primary and secondary endpoints from inclusion to low MAP target period and from low to high MAP target period (n = 26). P-values for the within-subject comparison of MAP using a multivariate repeated-measures analysis of variance (MANOVA). [file 13613_2021_925_MOESM1_ESM.docx]

**Supplemental table 1. Blood and urine parameters according to the order of administration of the pressure regimen** **(n=26)**

| **Variables** | **At inclusion** | | **Low-MAP target period** | | **High-MAP target period** | | **P values for Group-MAP interaction** |
| --- | --- | --- | --- | --- | --- | --- | --- |
|  | **Group A** | **Group B** | **Group A** | **Group B** | **Group A** | **Group B** |  |
| **Primary Endpoint** |  |  |  |  |  |  |  |
| Creatinine clearance (mL/mn)^a^ | 32 (21) | 37 (27) | 23 (24) | 40 (36) | 43 (35) | 76 (44) | 0.1 |
| **Secondary Endpoints** |  |  |  |  |  |  |  |
| Serum creatinine (µmol/L) | 162 (81) | 119 (75) | 175 (92) | 123 (69) | 166 (87) | 111 (63) | 0.5 |
| Urine creatinine (mmol/L) | 6.8 (3.4) | 7.8 (5.7) | 6.4 (3.5) | 9 (7.9) | 6 (3.2) | 7.1 (5.1) | 0.2 |
| Urine output (mL/kg/h) | 0.6 (0.3) | 0.8 (0.6) | 0.7 (0.4) | 0.9 (0.7) | 0.8 (0.5) | 1.2 (0.8) | 0.3 |
| Urine sodium (mmol/L) | 40 (22) | 49 (38) | 39 (18) | 41 (34) | 48 (25) | 51 (28) | 0.8 |
| Serum osmolality (mOsm/Kg) | 310 (17) | 305 (19) | 309 (17) | 304 (20) | 307 (14) | 305 (18) | 0.2 |
| Urine osmolality (mOsm/Kg) | 395 (115) | 488 (158) | 398 (125) | 518 (212) | 396 (109) | 485 (136) | 0.1 |
| Proteinuria (g/L) | 1.3 (0.8) | 1.2 (1) | 1.1 (0.7) | 1.1 (0.4) | 1.3 (0.9) | 1.2 (0.5) | 0.9 |
| Urine–plasma creatinine ratio | 59 (56) | 93 (86) | 58 (79) | 101 (91) | 53 (52) | 90 (87) | 0.8 |
| Fractional Excretion of Sodium | 1 (1.1) | 0.8 (1) | 1.4 (2) | 0.7(1.1) | 1.5 (1.6) | 0.8 (0.9) | 0.8 |
| Fractional Excretion of Urea | 32 (19) | 30 (14) | 33 (12) | 31 (15) | 36 (19) | 36 (15) | 0.2 |

Data are expressed as the mean (SD).

Group A: from high to low MAP target; Group B: from low to high MAP target

^a^calculated using the UV/P formula.

P-values for the within-subject comparisons between the low and the high-MAP target period a multivariate repeated-measures analysis of variance (MANOVA) including the order of assignment of MAP regimes and the KDIGO stage at inclusion as factors.

MAP, mean arterial pressure; KeGFR, kinetic estimated glomerular filtration rate

**Supplemental Table 2. Mean individual difference in blood and urine parameters according to KDIGO stage (KDIGO 1 patients (n=9) and KDIGO 2-3 patients (n=17))**

| **Variables** | **At inclusion** | | **Low-MAP target period** | | **High-MAP target period** | | **P values for Group-KDIGO interaction** |
| --- | --- | --- | --- | --- | --- | --- | --- |
|  | **KDIGO 1** | **KDIGO 2-3** | **KDIGO 1** | **KDIGO 2-3** | **KDIGO 1** | **KDIGO 2-3** |  |
| **Primary Endpoint** |  |  |  |  |  |  |  |
| Creatinine clearance (mL/mn)^a^ | 48 (27) | 27 (19) | 53 (42) | 21 (17) | 82 (36) | 46 (40) | 0.9 |
| **Secondary Endpoints** |  |  |  |  |  |  |  |
| Serum creatinine (µmol/L) | 105 (67) | 161 (81) | 99 (60) | 178 (84) | 98 (62) | 163 (81) | 0.04 |
| Urine creatinine (mmol/L) | 5.8 (3.8) | 8 (4.9) | 6.2 (3.6) | 8.4 (6.9) | 4.6 (2) | 7.5 (4.6) | 0.8 |
| Urine output (mL/kg/h) | 0.9 (0.2) | 0.5 (0.5) | 1.1 (0.6) | 0.6 (0.4) | 1.4 (0.5) | 0.8 (0.6) | 0.5 |
| Urine sodium (mmol/L) | 62 (34) | 35 (24) | 47 (34) | 36 (22) | 67 (31) | 41 (18) | 0.4 |
| Serum osmolality (mOsm/Kg) | 303 (18) | 310 (18) | 301 (15) | 310 (19) | 299 (11) | 310 (16) | 0.6 |
| Urine osmolality (mOsm/Kg) | 429 (131) | 440 (150) | 491 (176) | 437 (184) | 479 (158) | 405 (99) | 0.2 |
| Proteinuria (g/L) | 0.9 (0.5) | 1.4 (1) | 0.8 (0.3) | 1.3 (0.6) | 1 (0.4) | 1.4 (0.8) | 0.6 |
| Urine–plasma creatinine ratio | 77 (75) | 74 (73) | 93 (99) | 70 (80) | 71 (62) | 70 (77) | 0.1 |
| Fractional Excretion of Sodium | 1.1 (1.1) | 0.8 (1) | 0.9 (1.2) | 1.2 (1.9) | 1.4 (1) | 1.2 (1.5) | 0.8 |
| Fractional Excretion of Urea | 40 (14) | 26 (17) | 37 (9) | 29 (15) | 44 (13) | 32 (18) | 0.8 |

Data are expressed as the mean (SD).

^a^calculated using the UV/P formula.

P-values for the within-subject comparisons between the low and the high-MAP target period a multivariate repeated-measures analysis of variance (MANOVA) including the order of assignment of MAP regimes and the KDIGO stage at inclusion as factors.

MAP, mean arterial pressure; KeGFR, kinetic estimated glomerular filtration rate

**Supplemental Figure 1. Individual profile of primary and secondary endpoints.** Box plot (median values, inter-quartile range) and individual variations of primary and secondary endpoints from inclusion to low MAP target period and from low to high MAP target period (n=26). P-values for the within-subject comparison of MAP using a multivariate repeated-measures analysis of variance (MANOVA).
